# Retinal Vein Occlusion and Pregnancy, Pre-Eclampsia, and Eclampsia: The Results from a Nationwide, Population-Based Study Using the National Claim Database

**DOI:** 10.1371/journal.pone.0120067

**Published:** 2015-03-16

**Authors:** Sang Jun Park, Nam-Kyong Choi, Kyung Ha Seo, Kyu Hyung Park, Se Joon Woo

**Affiliations:** 1 Department of Ophthalmology, Seoul National University College of Medicine, Seoul National University Bundang Hospital, Seongnam, Republic of Korea; 2 Medical Research Collaborating Center, Seoul National University Hospital and Seoul National University College of Medicine, Seoul, Republic of Korea; 3 Institute of Environmental Medicine, Seoul National University Medical Research Center, Seoul, Republic of Korea; University of Barcelona, SPAIN

## Abstract

**Objective:**

To investigate the incidence of retinal vein occlusion (RVO) in pregnant women and in the subpopulation of pregnant women with pre-eclampsia/eclampsia compared to that in the age-matched general female population to determine if there is increased risk of RVO in pregnancy.

**Design:**

Nationwide population-based retrospective study using data entered into the Korean national health claims database from 2007 to 2011.

**Setting and Participants:**

Of the incident RVO cases in the database, RVO cases that occurred during the pregnancy-associated period, which spanned a 52-week period from 40-weeks-before to 12-weeks-after childbirth, were identified. Of these cases, the presence of pre-eclampsia/eclampsia was determined.

**Main Outcome and Measure:**

The standardized incidence ratios (SIRs) of RVO in the general pregnant population and in the pregnant population with pre-eclampsia/eclampsia were determined with respect to the age-matched general female population.

**Results:**

Pregnancy-related RVO was identified in 33 cases from the 1.8 million women who experience childbirth during the study period, while the expected number of cases calculated by the direct standardization to the age-matched general population was 113. Of the 33 patients, 12 patients (36.4%) had pre-eclampsia or eclampsia. The SIR for the general pregnant population in reference to the age-matched general female population was 0.29 (95% CI, 0.20–0.41). In contrast, the SIR for the pregnant population with pre-eclampsia/eclampsia in reference to the age-matched general female population and the age-matched general pregnant population was 67.50 (95% CI, 34.88–117.92) and 246.50 (95% CI, 127.37–430.59), respectively.

**Conclusions and Relevance:**

The results suggest that pre-eclampsia/eclampsia is a risk factor for RVO, while pregnancy itself may not be a risk factor for RVO.

## Introduction

Pregnancy and the subsequent puerperium represent hypercoagulable states with an increase in thrombin levels and pro-coagulant factors and a decrease in the levels of the endogenous anticoagulant protein S. The effects of progesterone on the blood vessel walls result in venous stasis, even in normal pregnancy [1, 2]. The increased risk of thrombotic disorders during pregnancy and puerperium has been consistently reported [1, 3–6]; therefore, an increased risk of vision-threatening thrombotic conditions such as retinal vein occlusion (RVO) might also be present. RVO is the second most frequently occurring retinal vascular disease and one of the foremost sight-threatening conditions [7–10]. There have only been a few reports of pregnancy-related RVO cases: 3 during normal pregnancy [11–13], 1 during pregnancy with pre-eclampsia [14], and 1 postpartum case following pregnancy with pre-eclampsia [15]. Despite this limited evidence, pregnancy is generally considered a risk factor for RVO [16].

Recently, we conducted a nationwide epidemiologic study regarding the RVO incidence in Korea using the national health claims registry [10]. Based on the gender-based incidence within each 10-year age group, we observed a higher RVO incidence in women in addition to a higher RVO incidence ratio (women:men) in those aged 20–29 years compared to those aged <20 years or ≥30 years.

Because of the known association between pregnancy and thromboembolic events, we hypothesized that pregnancy may have influenced the incidence of RVO in women aged 20–29 years in the previous study, an age at which pregnancy frequently occurs. Therefore, as a follow-up to our previous study, we used the same data set to evaluate the association between pregnancy and RVO [10].

## Methods

We accessed health claims from the years 2007–2011 in the database of the national Health Insurance Review and Assessment (HIRA) service of Korea; results from this data set have also been published previously [10]. In brief, the HIRA reviews all of the health claims in Korea, including those submitted through the Korean National Health Insurance scheme, which covers 97% of the population, and the other available medical assistance programs (i.e., the Medical Assistance Program and Medical Care for Patriots and Veterans Affairs Scheme), which cover the remaining 3% of population. Subsequently, the HIRA database stores data for the entire Korean population and their medical claims [10, 17, 18] and includes diagnoses, procedures, prescription records, and demographic information. Patients in the HIRA can easily be identified by their unique Korean Resident Registration Number, which is assigned to each Korean resident at birth; this ensures no duplication or omission when accessing the data. Public access to the HIRA database is not allowed; restricted access to the HIRA database is allowed only after approval by the HIRA Deliberative Committee for studies that are conducted for the common good [10, 17, 18]. The HIRA Deliberative Committee approved conditional use of data from the years 2007–2011 in the HIRA database for this and our previous study [10]. The provided database was de-identified by the HIRA before we could access the database. The institutional review board (IRB) of the Seoul National Bundang Hospital approved the study (IRB No. X-1211/177-901), which was conducted in accordance with the Declaration of Helsinki.

**Fig 1:**
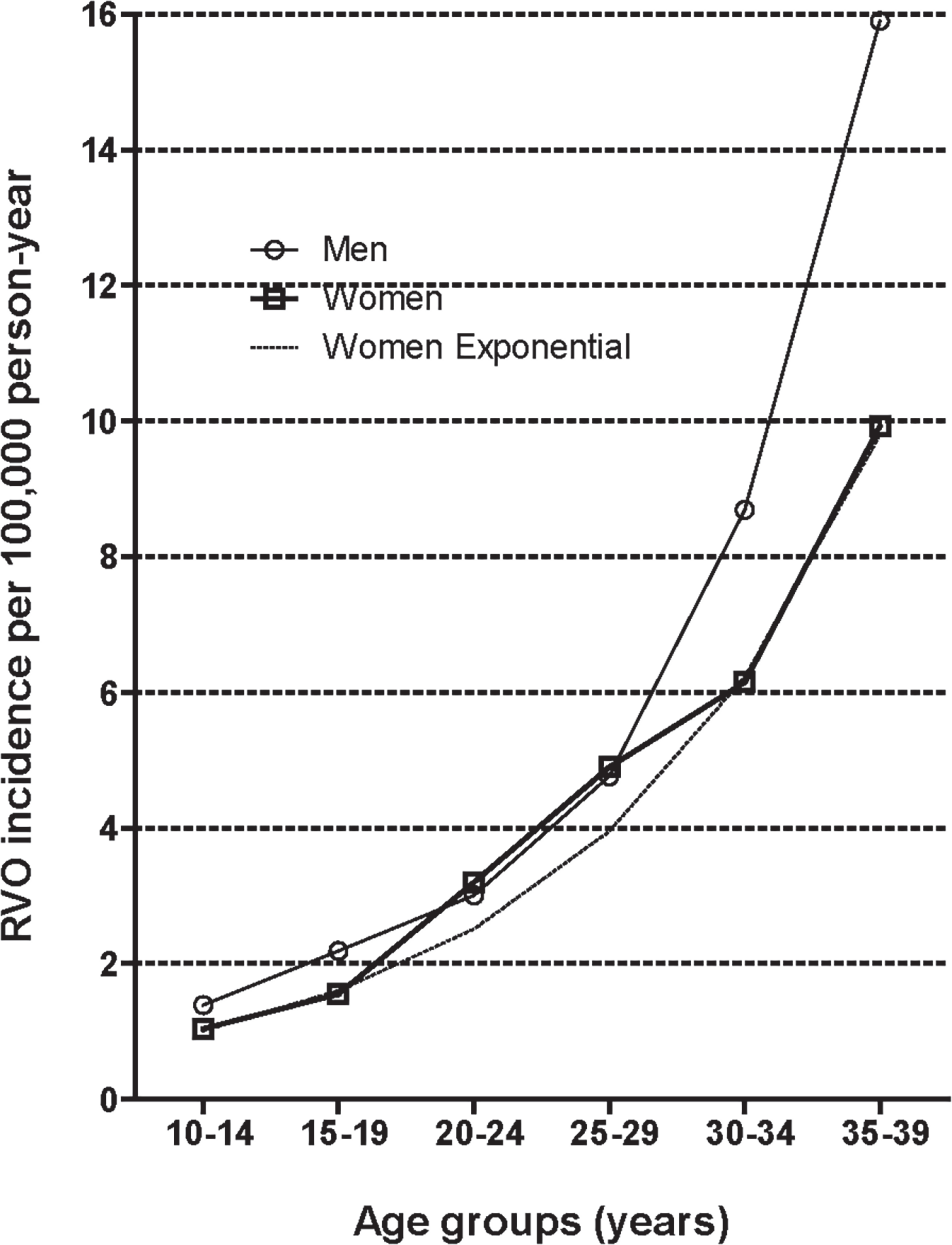
Annual incidence (2008–2011) of retinal vein occlusion (RVO) in Korea

We identified female cases of RVO using the first occurrence of an RVO diagnostic code (H34.8) according to the Korean Classification of Disease, sixth edition (KCD-6), a version of the International Classification of Diseases-10 adapted for the Korean health care system using the same data set of our previous study, which had only the data for patients with at least diagnosis of RVO [10]. The date of the earliest claim with an RVO diagnostic code was defined as the index date and was considered the incident time. To remove any potential preexisting cases of RVO, we excluded cases that had a RVO diagnostic code during the first year of the study period (2007). Our previous experiences with the data indicated that 1 year was sufficient to identify the disease-free period [10, 18]. The data from the Population and Housing Census conducted in 2010 were used to define the population of Korea, considered the population at risk of RVO (Korean Statistical Information Service, available at http://kosis.kr, accessed November 19, 2013). The frequency and estimated incidence rates of RVO in women in the entire general Korean population during the study period, classified according to age groups, are provided in **Fig. 1 and Table 1**; these data have previously been reported [10].

**Table 1:** Retinal vein occlusion (RVO) incidence in the general and pregnant Korean female population (2008–2011).

Age (years)	Entire population of Korean women		Sub-population of pregnant Korean women			
	RVO patients (n)	Incidence of RVO^a^ (per 100,000 person/years)	Population at risk^b^	Expected RVO (n)	Observed RVO (n)	SIR^c^ (95% CI)^d^
≤19	100	1.55	9,826	0.2	1	6.57 (0.17–36.58)
20–24	183	3.20	99,641	3.2	0	0.00 (0.00–0.94)
25–29	340	4.90	605,923	29.7	7	0.24 (0.09–0.49)
30–34	450	6.15	803,139	49.4	18	0.36 (0.22–0.58)
35–39	810	9.93	250,833	24.91	6	0.24 (0.09–0.52)
40–44	1,544	18.74	29,260	5.5	1	0.18 (0.00–1.02)
≥45	2,942	36.25	817	0.3	0	0.00 (0.00–10.12)
Sum	11,351		1,799,439	113.1	33	0.29 (0.20–0.41)

^a^The frequency and estimated incidence of RVO in the general population have already been published. (RVO Reference) While the data were provided for age ranges 15–19 years and 45–49 years, we reclassified these as ≤19 years and ≥45 years, respectively, as in our previous study.

^b^The population at risk for the pregnant population was defined as the number of women experiencing childbirth during the study period (2008–2011).

^c^The SIR was derived as the ratio of observed to expected RVO incidence in pregnant women during the 52-week period from 40 weeks before to 12 weeks after childbirth.

^d^The 95% confidence interval (CI) was calculated based on the Poisson distribution.

Of the cases with incident RVO, we identified pregnancy-related cases using 2 methods. First, we used a strict definition of pregnancy; we identified cases that experienced childbirth, excluding pregnancies with an abortive outcome. Childbirth was identified by the associated surgical codes in the health claims, including those for all delivery types: vaginal delivery, cesarean delivery, and all other childbirth-related procedures/surgeries. As the Korean insurance systems cover all pregnancy-related medical access, Korean women rarely undergo pregnancy and childbirth without utilization of health care providers. Second, we used a more lax definition of pregnancy in order to capture pregnancies with an abortive outcome; we identified patients with a claim containing at least 1 of the pregnancy-related diagnostic codes (KCD-6; XV. pregnancy, childbirth, and the puerperium [O00-O99]) during the study period. The pregnancy-associated period was defined as the period spanning a 52-week period from 40 weeks before to 12 weeks after childbirth. Then, we identified the pregnancy-related RVO cases using an index date during the pregnancy-associated period. In addition, we collected data regarding pregnancy-related systemic comorbidities during the pregnancy-associated period using the diagnostic codes for the following: [1] edema, proteinuria, and hypertensive disorders in pregnancy, childbirth, and puerperium (O10-O16); [2] venous complications in pregnancy (O22); and [3] diabetes mellitus (DM) (O24). The observed RVO incidence rate in the pregnant population was calculated as the number of people who developed RVO divided by the population at risk. The pregnancy-associated period, which included 52 weeks (1 year), and RVO incidence are reported as “per person-years”, similar to our previous study [10].

**Fig 2:**
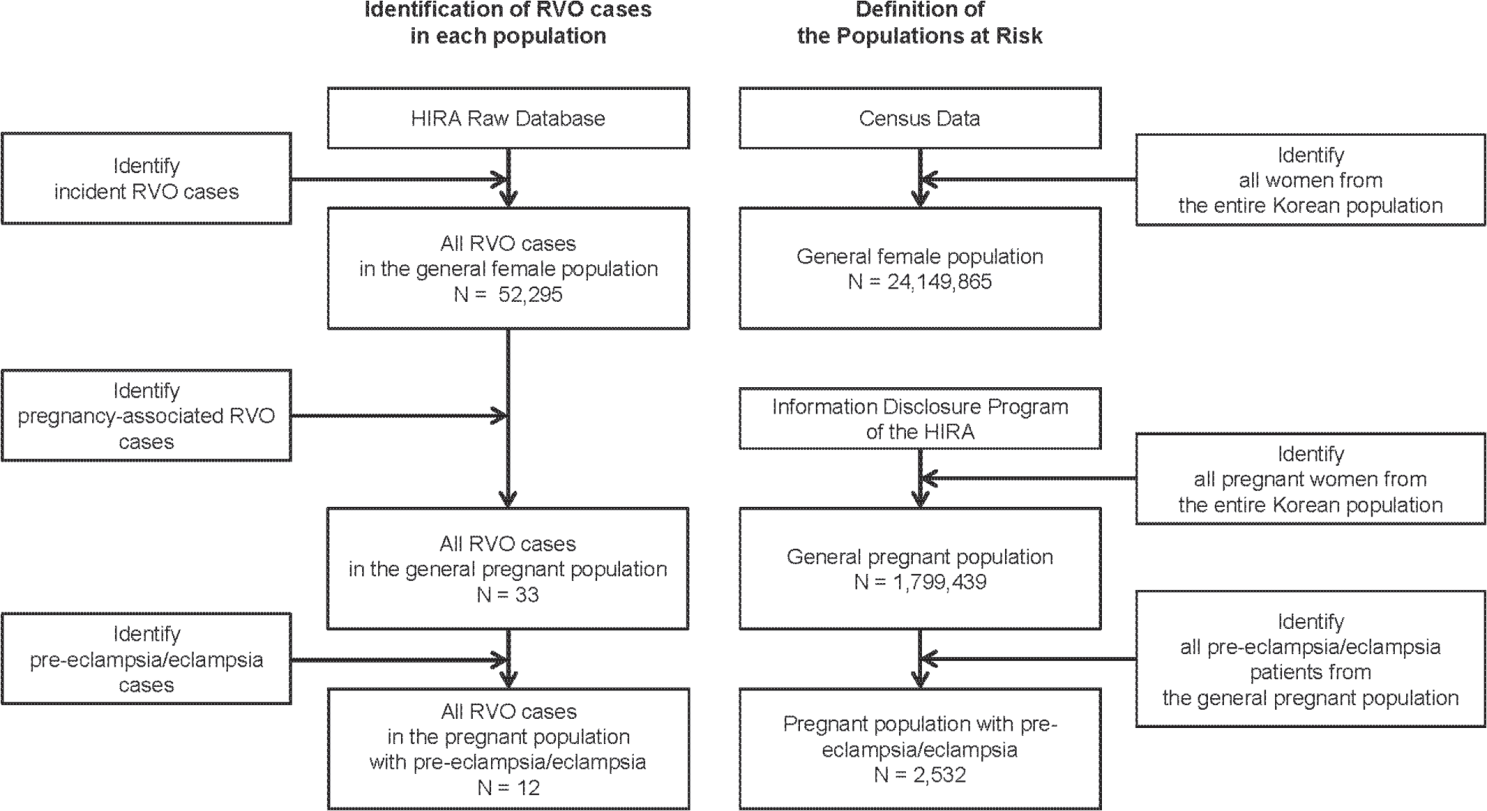
Flowchart regarding the identification of retinal vein occlusion (RVO) cases and the definition of each population at risk. Footnote: HIRA = the national Health Insurance Review and Assessment service of Korea; Census data = the Population and Housing Census conducted in 2010 in Korea; The information disclosure program of the HIRA provides the number of patients who were diagnosed with specific diseases and/or underwent procedures during a specific period, according to 5-year age groups, and does not provide information about health claims.

The population at risk was defined as the sample of women that experienced childbirth among the general population during the study period, which was estimated using the information disclosure program provided by the HIRA, which does not require approval by the Deliberative Committee. The information disclosure program provides the number of patients who were diagnosed with specific diseases and/or underwent procedures during a specific period, according to 5-year age groups, and does not provide information about health claims. According to this program, 1,799,439 Korean women experienced childbirth during the study period (**Table 1**). In addition, the observed RVO incidence rate in the women who experienced pre-eclampsia/eclampsia, which results in hyper-coagulopathy and could potentially cause pregnancy-related RVO, was calculated in the same way. The population at risk was defined as the sample of patients who experienced pre-eclampsia/eclampsia (n = 2,532) in the entire pregnancy-related population during the study period, which was also obtained from the information disclosure program of the HIRA. The process of identifying RVO cases and the definition of each population at risk are provided in **Fig. 2**.

We compared the age and prevalence of pregnancy-related systemic comorbidities between the women with pre-childbirth RVO and those with post-childbirth RVO using Chi-square tests. Then, we compared the incidence rate of RVO in the general pregnant population to that in the general female population using the appropriate standardization method [19]. Setting the general pregnant population as the reference population, the expected incidence cases for each age group of the general pregnant population were calculated by multiplying the age-specific incidence rates of the general female population and the number of persons in each age group of the general pregnant population, which was ascertained as described in the previous paragraph. After adding the number of expected incident cases from all ages, the age-adjusted (expected) incidence rate of RVO in the general pregnant population was estimated by dividing the total number of expected incident cases by the general pregnant population. The standardized incidence ratio (SIR) was derived as the ratio of the observed incidence rate to the expected incidence rate of RVO in the general pregnant population. We also compared the incidence rate of RVO in the pregnant population with pre-eclampsia/eclampsia to that in the general female population and to that in the general pregnant population by the same set of analyses. The 95% confidence intervals (CIs) were calculated based on the Poisson distribution. We used SAS, version 9.3 (SAS Institute, Inc, Cary, North Carolina) for all analyses. A P value of < 0.05 was considered to indicate statistical significance.

## Results

According to the strict definition of pregnancy, there were 33 pregnancy-related RVO patients with a mean age of 31.79 ± 4.61 years (range: 17–43 years). Out of the 33 pregnancy-related RVO patients, 14 patients had an index date before childbirth, 18 patients had an index date after childbirth, and 1 patient had an index date on the same day of childbirth (**Table 2**). No additional RVO cases were identified using the lax definition of pregnancy, i.e., in those with an abortive outcome.

**Table 2:** Characteristics of women with pregnancy-related retinal vein occlusion (RVO) in Korea (2008–2011).

ID	Age (years)	RVO incidence related to day of childbirth (weeks)^a^	Pregnancy-related systemic complications
1	26	−40	Complicating/maternal HTN, DM
2	39	−37	None
3	35	−36	None
4	38	−35	DM
5	34	−32	None
6	32	−27	Pre-eclampsia, HELLP syndrome
7	38	−24	Pre-eclampsia, DM
8	43	−22	DM
9	32	−18	DM
10	25	−17	None
11	31	−16	None
12	30	−10	Pre-eclampsia
13	34	−5	None
14	28	−3	None
15	33	0	Severe pre-eclampsia
16	27	2	None
17	32	2	Severe pre-eclampsia
18	39	2	Severe pre-eclampsia
19	30	3	Severe pre-eclampsia
20	31	3	Pre-eclampsia
21	32	3	None
22	33	3	Severe pre-eclampsia
23	37	3	Eclampsia
24	35	5	None
25	36	5	Pre-eclampsia
26	17	6	None
27	36	6	DM
28	37	8	None
29	31	9	None
30	28	10	None
31	31	10	None
32	32	10	DM
33	28	12	Moderate pre-eclampsia

^a^Negative values indicate before childbirth, 0 indicates the day of childbirth, and positive values indicate after childbirth.

RVO, retinal vein occlusion; HTN, hypertension; DM, diabetes mellitus; HELLP, hemolysis, elevated liver enzymes, low platelet count

**Fig 3:**
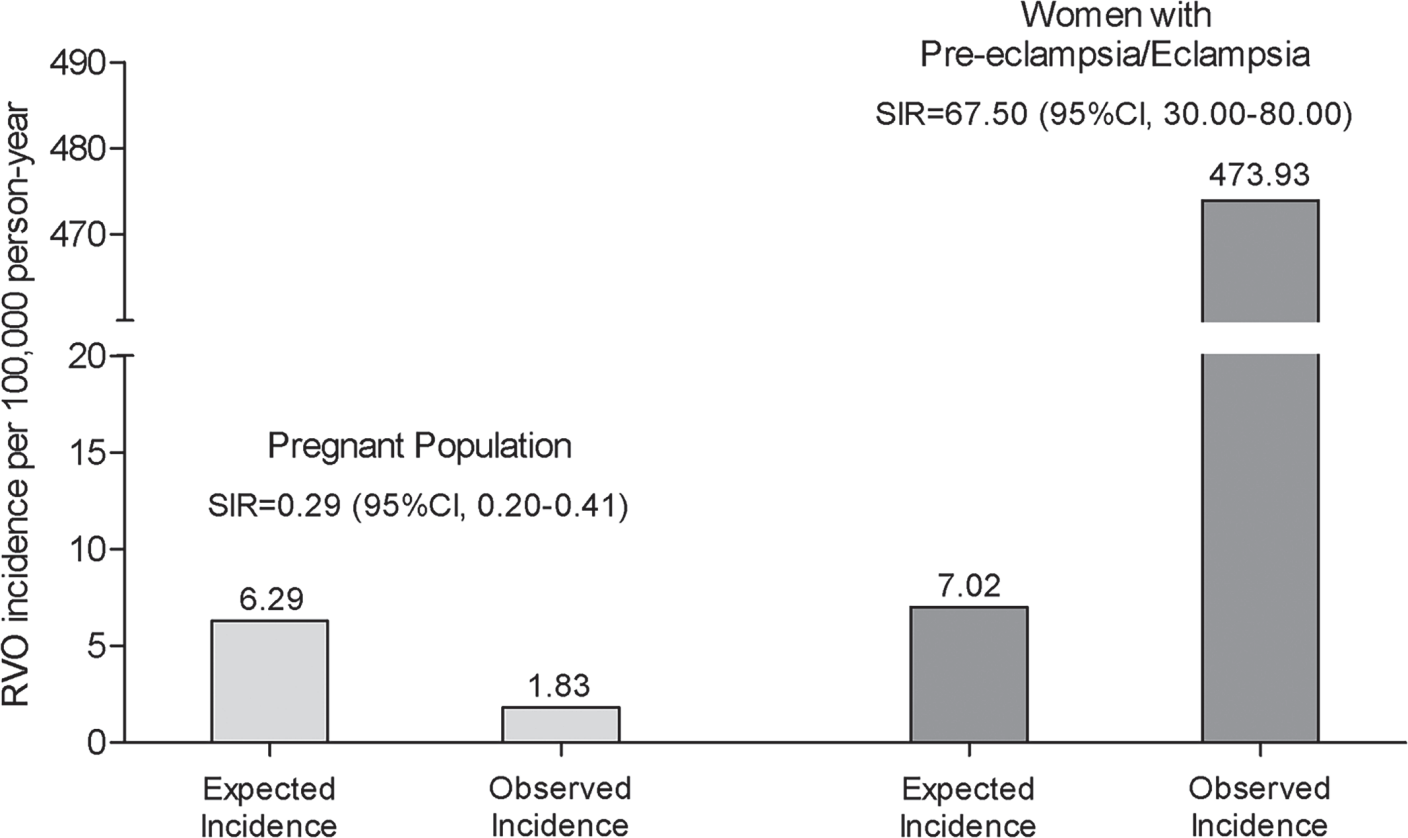
Retinal vein occlusion incidences in a sample of pregnant women and women with pre-eclampsia/eclampsia. Footnote: Expected incidences were estimated using proper standardized methods based on the general female population. Standardized incidence ratios (SIRs) are the ratio of observed to expected incidence of retinal vein occlusion. The 95% confidence intervals (CIs) were calculated using the Poisson distribution.

The number of expected pregnancy-related RVO cases was approximately 113, resulting in an expected incidence rate of 6.29 (95% CI, 5.13–7.44) per 100,000 person-years, while the observed incidence rate was considerably lower at 1.83 (95% CI, 1.21–2.46) per 100,000 person-years (**Table 1** and **Fig. 3**). The SIR was 0.29 (95% CI, 0.20–0.41), indicating that the incidence of RVO was 70% lower in the pregnant women compared to the general female population, which was statistically significant.

Of the 33 pregnancy-related RVO patients, 18 patients (54.5%) had pregnancy-related systemic comorbidities during the pregnancy-related period, including complicating hypertension; pre-eclampsia; hemolysis, elevated liver enzymes, and low platelet count (HELLP) syndrome; eclampsia; and DM (**Table 2**). Of the 18 patients, 12 patients had pre-eclampsia or eclampsia, while 5 patients had only DM without other complications. The observed RVO incidence rate among the pregnant population with pre-eclampsia/eclampsia was 473.93 (95% CI, 205.78–742.09) per 100,000 person-years, compared to the expected RVO incidence of 7.02 (95% CI, 0–39.66) per 100,000 person-years (**Fig. 3**). The corresponding SIR was 67.50 (95% CI, 34.88–117.92). In addition, the SIR of the pregnant population with pre-eclampsia/eclampsia in comparison to the general pregnant population was 246.50 (95% CI, 127.37–430.59).

There was no difference in age or the prevalence of pregnancy-related systemic comorbidities between the women with pre-childbirth RVO and those with post-childbirth RVO; the mean ages were 33.21 ± 5.14 and 31.78 ± 5.02 (*P* = 0.433), respectively, and the prevalence of pregnancy-related systemic comorbidities was 50.0% and 55.6% (*P* = 1.00), respectively.

## Discussion

The incidence of RVO related to pregnancy was significantly lower than that of the general female population of the same age, with only 33 cases of approximately 1.8 million deliveries (SIR, 0.29). This was contrary to our expected outcomes based on the higher RVO incidence in women than men in the primary reproductive age range of 20–29 years that we previously observed [10]. (**Fig. 1**) Furthermore, only a small number of RVO cases remained after excluding the patients with systemic comorbidities such as eclampsia/preeclampsia, which may significantly alter hemodynamics during pregnancy and are already considered a risk factor for RVO [14, 15, 20]. Therefore, our study suggests that normal pregnancy is not a risk factor for the development of RVO, but may instead be a protective factor.

Pregnancy has been considered a hypercoagulable state not only in patients with hereditary thrombophilia and/or pre-eclampsia/eclampsia but also in normal pregnancy, which is associated with acquired changes in hemostatic factors including plasma fibrinogen, factor VIII, protein C, protein S, and platelets [3]. The incidence of venous thromboembolism during the pregnancy-associated period is 6 times greater than that in non-pregnant women, particularly during the third trimester and postpartum period [21]. Several cases of RVO in pregnancy have been reported, primarily in pregnancy with pre-eclampsia/eclampsia but also in normal pregnancy; therefore, it is generally assumed that normal pregnancy and the associated hypercoagulable state may induce RVO [2, 11, 13–15, 20, 22]. However, the results of the present study suggest that pregnancy does not increase, but perhaps decreases, the risk of RVO.

There are several possible explanations for the low occurrence of pregnancy-related RVO observed in our study. First, the use of the entire population of pregnant women as the population at risk may have resulted in bias, namely the ‘healthy mother effect’. Women who decide to become pregnant may be healthier than those who choose not to become pregnant; further, pregnant women may be more likely to focus on health care during the pregnancy [23]. Moreover, as the Korean health insurance systems provide all prenatal care to pregnant women through the maternity clinics, most pregnant women in Korea undergo not only monthly regular prenatal obstetric checkups but also regular medical checkups that include blood pressure, blood sugar levels, anemia, and other measurements to detect all possible pregnancy-related complications. As a result, the RVO incidence may be lower than that of the general population owing to regular monitoring of risk factors and appropriate health care. Second, the hypercoagulable state in pregnancy may not be sufficient to develop RVO in young women unlike deep vein thrombosis (DVT) of the lower extremities, which occurs more frequently in pregnant women compared to age-matched general female population [1, 3, 21]. Although the pathophysiology of RVO has not been fully elucidated, RVO is thought to be an arterial disease; the atherosclerotic retinal artery compresses the adjacent retinal vein, causing hemodynamic changes, which, in turn, lead to the formation of thrombi [10]. Pregnancy itself may not result in stasis and endothelial injury of the retinal vein, which may be crucial to developing RVO. Furthermore, all reported pregnancy-related RVO cases have been central RVO (CRVO) [11–15, 22], indicating that CRVO-specific pathophysiology may be present during the pregnancy-associated period. Previous studies have suggested that an anatomical variation that affects the central retinal vein and its available tributaries at the level of, or at the posterior part of the lamina cribrosa may cause CRVO [7, 24]; the hypercoagulable state in pregnancy may cause CRVO in women with this anatomical variation, while the sole presence of the hypercoagulable state in pregnancy does not increase the risk of RVO.

In addition, we did not detect any RVO cases in pregnancy with an abortive outcome. All of the reported pregnancy-related RVO cases have occurred at or after the sixth month of pregnancy [11–15], and pregnancy-related thrombophilic events occur primarily in the third trimester [1, 3, 21]. Naturally terminated pregnancies that occur between 12 and 22 weeks of pregnancy represent only 4% of pregnancy loss, and late loss after 25 weeks of gestation is quite rare [25].

As expected, pre-eclampsia/eclampsia was associated with a significantly higher incidence of pregnancy-related RVO. Pre-eclampsia/eclampsia is characterized by poor placental perfusion, resulting in endothelial dysfunction [4, 15] and related retinal vascular abnormalities that may contribute to an increased RVO incidence, such as arteriolar narrowing, cotton wools spots, hemorrhages, and Elschnig spots [1, 2, 15]. To the best of our knowledge, this is the first report of the SIR of RVO in pregnant women with pre-eclampsia/eclampsia compared to the general population and the entire pregnant population, and the results suggest that particular attention should be paid to vision health in patients with pre-eclampsia/eclampsia.

Moreover, DM is widely accepted as a risk factor for RVO, especially CRVO [26–28]. Gestational DM occurs in approximately 1.8–6.9% of pregnant women, and DM that is present prior to pregnancy may deteriorate during pregnancy [2, 29]. Therefore, DM during pregnancy may play a role in the development of RVO. Unfortunately, we could not estimate the association between DM and RVO because the database had only claims of patients diagnosed as RVO at least once and did not include all of the claims with DM diagnostic codes. Instead, we were only able to present the number of patients with DM (21.2%) among the 33 pregnancy-related RVO cases, indicating that DM may play a partial role in RVO that occurs during pregnancy.

Based on the present results, pregnancy cannot explain why we previously observed an RVO incidence that was higher than expected for women and higher than that of men of the same age, in those aged 20–29 years [10]. As an alternative explanation, oral contraceptives could have contributed to the incidence in this age group [30–32]. As oral contraceptives are categorized as over-the-counter drugs in Korea, data regarding their use were not available in the database. Second, the military population may be healthier than the rest of the population (healthy soldier effect) [33], which might explain why women in this age group were more likely to have RVO than men of the same age. Korean men are obligated to actively serve in the military for at least 2 years with an additional 8 years in the reserves, while Koran women have no obligation regarding military service.

This study has certain limitations. First, we could not conduct a thorough analysis adjusting for all relevant covariates (e.g., systemic comorbidities such as hypertension, DM, or dyslipidemia) to estimate the association between RVO and pregnancy. We were not allowed to extract data for the control population that did not have RVO-related claims from the HIRA database [10, 17], and the information disclosure program of the HIRA provided only the number of patients with pregnancy and pre-eclampsia/eclampsia, without the details of other potential risk factors. However, the lack of covariates might not have significantly influenced the estimated association between RVO and pregnancy as indicated by the statistically significant 95% CI for the SIR; also, women aged 20–29 years are likely to have a low prevalence of systemic chronic diseases that would have been included as relevant covariates. In addition, we extracted all of the diagnostic codes for systemic co-morbidities during pregnancy and the postpartum period in the RVO patients and estimated the SIR for the pregnant population with pre-eclampsia/eclampsia. Second, the present study is not immune to the limitations inherent to studies using the health claims database [10, 17]. The diagnostic codes in the claims data may not be accurate and could not be validated by reviewing medical records. Further, we could not assess the severity, extent, and type of RVO nor the degree of the hypercoagulable state in each patient using laboratory data. Moreover, although we could not find any claims with diagnostic codes for both a miscarriage and RVO, some pregnancies with an abortive outcome might not have been claimed, resulting in an underestimation of the occurrence of pregnancy-related RVOs. Third, owing to the data available in our data set, we could not evaluate other pregnancy-related thrombophilic complications as DVT.

In the present study, using data from the national Korean health claim database, we were able to determine that normal pregnancy is not a risk factor for RVO, with a significantly lower incidence of RVO in the pregnancy-associated period than in the general female population. In contrast, the presence of pre-eclampsia/eclampsia during pregnancy is associated with a significantly higher incidence of RVO than in both the general pregnant population and the general female population. The present study suggests that pre-eclampsia/eclampsia is a risk factor for RVO, while normal pregnancy may not be a risk factor for RVO.
